# Harnessing Workplace Ostracism: Unleashing Proactive Behavior through Work Focus and Visionary Leadership

**DOI:** 10.3390/bs14070566

**Published:** 2024-07-04

**Authors:** Guang Xu, Shan Liu, Jie Zhong, Haiyan Yang

**Affiliations:** 1 School of Economics and Management, Harbin Normal University, Harbin 150025, China; gregxu@hrbnu.edu.cn (G.X.); haiyan@hrbnu.edu.cn (H.Y.); 2 School of Business Administration, South China University of Technology, Guangzhou 510642, China; liushan@hrbnu.edu.cn; 3 School of Economics and Management, Tsinghua University, Beijing 100084, China

**Keywords:** workplace ostracism, work focus, visionary leadership, proactive behavior, regulatory focus theory

## Abstract

Differing from prior studies which explored workplace ostracism’s negative impacts, in this study, we try to explore ways to mitigate and harness workplace ostracism to encourage proactive behavior. By drawing on regulatory focus theory, we propose that workplace ostracism can increase proactive behavior via enhanced promotion focus and prevention focus. We collected questionnaire data at multiple time points from employees in private enterprises in China, and a structural equation model was primarily used to test the proposed model. The results of the study indicate that workplace ostracism positively relates to work focus, which, in turn, improves employees’ proactive behavior. Visionary leadership only moderates the relationship between workplace ostracism and promotion focus. Employees who perceive high levels of visionary leadership exhibit an increase in promotion focus after experiencing workplace ostracism. This increase in promotion focus further enhances their proactive behavior. These research findings clarify the pathway and boundary conditions through which workplace ostracism positively influences proactive behavior. They also provide valuable insights for enterprises seeking to promote proactive behavior among employees.

*I can’t change the direction of the wind, but I can adjust my sails to always reach my destination*.

—Jimmy Dean

## 1. Introduction 

Some employees are often neglected and isolated, which is a common phenomenon in the workplace. Workplace ostracism widely exists in various types of organizations, which has great influence on both organizations and employees, and it has aroused concern in managers and researchers. Previous studies have primarily focused on the negative effects of workplace ostracism [1], such as increased emotional exhaustion [2], reduced creativity [3] and helping behavior [4], and diminished proactive behavior [5]. It is hard to perish ostracism. Although it is necessary to acknowledge the harmful nature of ostracism, understanding how individuals cope with and harness ostracism for personal growth becomes more crucial. Recent research has begun to shed light on the potential positive outcomes associated with workplace ostracism. For example, skilled emotional inhibition has been found to promote organizational learning [6], and individuals facing workplace ostracism can enhance their innovation performance [7]. Of course, as we emphasize the positive aspects of ostracism, we do not mean to endorse or encourage this phenomenon. Rather, the objective is to gain insights into the underlying mechanisms or conditional factors that can be utilized for personal and organizational development.

Proactive behavior not only encompasses task completion and self-improvement (intra-role behavior), but also extra-role behaviors that contribute to organizational development [8]. Focusing on proactive behavior as the dependent variable allows us to examine how individuals, despite experiencing exclusion, can still contribute positively to their work and the organization as a whole. Although Xu et al., (2017) used the self-verification theory to demonstrate that excluded employees exhibit an increase in helping behavior [9], they only indicated that employees adopt certain interpersonal mitigating behaviors to survive in an ostracism-prone workplace. Individuals are not always forced to engage in mitigating behaviors after experiencing workplace ostracism, but they may also initiate change for reasons such as wanting to demonstrate their self-worth or wanting to achieve certain goals. Therefore, the relationship between workplace ostracism and proactive behavior may be affected by work focus. Previous studies primarily treated work focus as a stable personal trait and explored its’ moderating role [10,11]. However, work focus will also be stimulated by certain situations [12]. This makes the research on the formation path of work focus insufficient. 

To make up for the above gap, in the theoretical framework of regulatory focus theory [12], we propose that workplace ostracism may improve proactive behavior via increased promotion focus and prevention focus. Workplace ostracism stimulates promotion focus by triggering a desire to prove one’s worth and regain acceptance, while it activates prevention focus by creating a sense of insecurity and the need to avoid further exclusion. As a result, individuals engage in proactive behaviors to overcome the negative effects of ostracism and achieve personal growth and goal attainment.

Regulatory focus theory suggests that leadership may modify followers’ regulatory focus [13]. Visionary leadership differs in the extent and frequency that these leaders refer to their organization’s long-term vision, that is, the collective self of what the organization should be in the future [14,15]. This leadership style is more relevant to workplace ostracism. When faced with ostracism, visionary leadership serves as a source of inspiration and makes employees care more about collective interests rather than personal loss. Visionary leaders make plans and take actions based on the espoused vision, which can help employees connect the present to the future self [14]. Additionally, visionary leadership not only creates an environment that encourages growth, achievement, and success, but also recognizes the need to address risks, maintain stability, and foster a secure work environment and creativity [16,17]. As such, visionary leadership can enhance the relationship between workplace ostracism and promotion focus and prevention focus at the same time.

We used the questionnaire survey method to conduct research in enterprises in China, Shandong, Guangdong at two time points. Then, we used the structural equation model (SEM) and PROCESS program to analyze the data, and we found that the results support our hypothesis. In summary, this research contributes to the literature in the following ways: Firstly, it enriches workplace ostracism’s positive aspects by exploring its positive relationship with proactive behavior. Secondly, it provides empirical support for the regulatory focus theory. Previous studies primarily treated work focus as a stable personal trait and explored its moderating role [10,11], while this study treats work focus as a psychological state. Additionally, prior research mostly thought that promotion focus and prevention focus contrast with each other [18]. We suggest that these two regulatory focuses can exist and be increased at the same time. Finally, we state that visionary leaders have a greater effect on promotion focus than prevention focus. 

## 2. Theoretical Overview and Hypotheses 

### 2.1. Regulatory Focus Theory 

Regulatory focus theory suggests that there are two separate regulatory focuses within each individual that are driven and motivated by them, namely promotion focus and prevention focus [12]. A high expectation of the ideal and final state motivates an individual’s promotion focus and encourages them to view situations in a gain/non-gain framework [12,19]. So, individuals focus on the ideal self and a vision of the good [20], whereas individuals with a high prevention focus are more concerned with responsibility and obligation, focusing on what they should accomplish and what others should expect of them [20]. They tend to view situations in a loss/non-loss framework [12] and avoid undesired outcomes. 

Both promotion focus and prevention focus are related to achieving goals, but the difference is that the two focuses help achieve goals by adopting different strategies [19,21,22]. The two focuses are independent of each other and can co-exist, so it is possible for an individual to have high levels for both focuses, or to have one high-level focus and one low-level focus, or two low levels of focus [22,23]. Both focuses can either be developed over time or induced by a situation. In a specific setting, such as the workplace [22], focus also becomes stable over time.

Based on the above theory, when faced with workplace ostracism, employees exhibit different strategies and orientations to deal with the situation, known as work focus. Work focus, comprising promotion focus and prevention focus, is a psychological state influenced by personality, situational factors, and work context [24]. According to regulatory focus theory, a high desire for goals stimulates promotion focus; insecurity and defensive states stimulate prevention focus. These two focuses are created to harness workplace ostracism to achieve goals while also mitigating the negative effects of environmental stress. Therefore, in order to cope with workplace ostracism, individuals will create two kinds of focus at the same time so as to achieve an increase in proactive behavior in different ways.

### 2.2. Workplace Ostracism and Work Focus

Workplace ostracism, characterized by neglect and isolation experienced by individuals in the workplace, has significant psychological implications for employees [1,25]. One of the positive effects of workplace ostracism is its influence on employees’ promotion focus. Individuals feel stress when ostracism affects their work. This difference requires immediate cognitive and behavioral adjustments to manage these differences and to adjust one’s work status [26]. In the face of exclusion and neglect, individuals may perceive an opportunity to demonstrate their abilities, skills, and potential to overcome negative experiences [27]. This perception focuses on potential gains and opportunities for advancement, which is in line with the promotion focus of pursuing positive outcomes [26]. Therefore, the opportunities demonstrated by workplace ostracism increase an individual’s desire to pursue positive outcomes. If individuals continue to perform well in spite of the pressure of ostracism, they may gain from the experience (e.g., resilience or competence recognition). At the same time, employees may also develop an emotional response to workplace ostracism, and it should be determined whether ostracism may arise due to jealousy towards others [28] or due to lower performance. 

According to the regulatory focus theory, this perception will help employees set a promotion goal, and promotion focus can be motivated by this desired goal. Therefore, based on the desire for self-improvement, when employees feel excluded in the workplace, they direct their attention towards the benefits that can be derived from the situation. They view the stress associated with ostracism as an opportunity for future development and enhanced job performance, thereby stimulating their promotion focus. Hence, we propose the following subhypothesis:

**H1a:**  *Workplace ostracism has a positive effect on promotion focus*.

Workplace ostracism can also have a degree of influence on prevention focus. The consensus among scholars is that workplace ostracism can negatively affect positive factors such as work engagement, helping behavior, and well-being [4,29,30]. The findings (e.g., potential negative outcomes and loss of resources) revealed by this negative impact are consistent with those regarding prevention focus [26]. Because of this negative information, employees may develop negative emotions. This should prompt employees to take a prudent, failure-averse approach to defusing negative emotions in a timely manner.

In addition to the two causes of jealousy and performance, workplace ostracism that is unrelated to work signifies a form of exclusion without specific reasons. An individual perceives this unjustified form of aggression as a source of loss of work resources. For example, in workplace ostracism, an employee loses emotional value [2] and is thus less productive. Such situations, categorized as “loss”, trigger employees’ prevention focus, leading individuals to prioritize safety and fulfilling their responsibilities [27]. They will seek to avoid the appearance of further negative outcomes as a result of not fulfilling their responsibilities. Guided by the need for self-preservation and security, individuals experiencing workplace ostracism are inclined to adopt a defensive mindset, focusing on the execution of their duties and obligations. They become cautious and meticulous, considering potential losses during the work process and anticipating negative outcomes upon task completion. According to the regulatory focus theory [12], situations can stimulate an individual’s regulatory focus; therefore, the defensive mindset and sense of responsibility brought about by workplace ostracism stimulates prevention focus in employees. Consequently, workplace ostracism induces employees’ prevention focus. Hence, we propose the following subhypothesis:

**H1b:**  *Workplace ostracism has a positive effect on prevention focus*.

### 2.3. The Mediating Role of Work Focus between Workplace Ostracism and Proactive Behavior

Proactive behavior is characterized by individuals taking initiative and performing anticipatory actions to address pressure and improve their circumstances [31]. Employees who experience workplace ostracism and develop a dual focus are likely to adopt different strategies to actively seek ways to enhance their performance, contribute to the organization, and elevate their social standing. Therefore, work focus enhances employees’ proactive behavior.

Employees who develop promotion focus are driven by their aspirations and positive ideals [15]. Whether the aim is to alleviate negative emotions or to improve oneself, promotion focus helps an individual achieve goals [26]. Since promotion focus primarily focuses on the positive aspects of work, the impending benefits and opportunities of workplace ostracism motivate employees’ promotion focus [32]. They will seek to realize their visions and ideals, leading them to take a proactive approach to achieving their goals. And employees tend to proactively implement risk-taking and exploratory strategies [33] to pursue their goals, such as expanding their resources, capabilities, and social circles while earning trust and attention among their colleagues [26]. This is also a means by which workplace ostracism can be mitigated. 

In particular, when individuals perceive a noticeable disparity between the desired goal and the current state, they proactively take action to minimize losses or maximize gains [34]. This is because the desire for goals and proactive strategies drives employees to be proactive and improve the current environment. Promotion focus increases employees’ sensitivity to the positive aspects of their environment [26]. So, even when employees feel excluded, they continue to enhance their skills, provide constructive suggestions for team operations, and assist the team in improving performance [35]. Regulatory focus theory suggests that people with promotion focus value the pursuit of “aspirational” goals and pursue these optimistically across a wide range of potential opportunities [23]. The urgent expectation of aspirational goals drives proactive employee behavior. Consequently, promotion focus induces employees to exhibit proactive behavior in order to achieve growth-oriented goals and proactively address challenges [31]. Based on these considerations, we propose the following hypothesis:

**H2a:**  *Promotion focus mediates the relationship between workplace ostracism and proactive behavior*.

Workplace ostracism triggers a perception of insecurity, leading individuals to prioritize their survival and security needs. In response to exclusion from the workplace, employees tend to adopt defensive and conservative strategies to prevent negative outcomes [21]. Therefore, prevention focus produces intangible changes in work role perceptions (e.g., changes in affective experiences) [36]. Their self-protective mechanisms drive them to take adaptive actions to improve their relationships with team members [37], resulting in an increase in proactive behavior, such as helping behavior [9], or enhancing their work effectiveness.

Proactive behavior typically involves individuals taking initiative and engaging in actions to achieve goals and minimize potential risks [38]. Prevention focus, on the other hand, is associated with a cautious and risk-averse mindset. These two concepts may seem contradictory at first glance.

However, it is important to note that proactive behavior can manifest in different ways. While one aspect of proactive behavior involves seeking opportunities and taking initiative, another aspect involves actively preventing or mitigating potential risks and problems [38]. In the context of workplace ostracism, individuals with prevention focus may engage in proactive behavior by taking steps to protect themselves from further harm and minimize the negative effects of ostracism. And, as prevention focus is concerned with fulfilling responsibilities and obligations, employees will be proactive in fulfilling their obligations to prevent workplace ostracism from being exacerbated by unfinished work. Therefore, prevention focus serves as a mediator between workplace ostracism and proactive behavior, and we propose the following subhypothesis:

**H2b:**  *Prevention focus mediates the relationship between workplace ostracism and proactive behavior*.

### 2.4. The Moderating Effect of Visionary Leadership 

According to the regulatory focus theory, individuals adjust their focus to meet the demands of leaders [13,39]. Employees are likely to align themselves with the leader’s expectations and reinforce both promotion focus and prevention focus. Stated differently, visionary leaders are likely to communicate and motivate team members to share their visions, so leaders and employees have goal congruence [40]. Goal congruence gives employees the motivation to pursue a better future.

High-level visionary leaders possess strategic planning skills and effective communication abilities that inspire employees to accept, identify with, and act upon their visions [41]. An effective vision can alleviate employees’ negative press and enhance job performance [15]. Explaining the significance of work to employees can improve their goal achievement rates [14]. Therefore, the level of communication is key to enhancing employee promotion focus. The demand for performance and goals further strengthens the inclination towards promotion focus. Thus, when employees feel the pressure of workplace ostracism, communication from visionary leaders is more likely to stimulate their desire to achieve growth goals. In addition, at the heart of visionary leadership is the creation and communication of a compelling vision. No other type of leadership addresses the construction and communication of a desired future state as directly as visionary leadership [40]. And leaders develop a vision that is perceived as inspiring and an end state that employees can accomplish. Visionary leadership may also enhance employees’ promotion focus. The encouragement and goal planning provided by visionary leadership encourage employees to identify growth opportunities from experiences involving pressure and actively pursue a better vision [42]. As a result, based on the alignment of achievement needs, promotion focus is reinforced, enabling better goal attainment. Based on the above reasoning, the following subhypothesis is proposed:

**H3a:**  *Visionary leadership moderates the relationship between workplace ostracism and promotion focus. The higher the degree of visionary leadership, the stronger the positive relationship between workplace ostracism and promotion focus*.

Visionary leaders can help employees connect the present to the future self by making plans and taking actions based on the espoused vision [14]. This can provide excluded employees with the motivation to change the status quo to achieve an ideal future, thereby promoting their desire to achieve their visions [16]. Because workplace ostracism can cause resource loss, employees may be confused. Thus, visionary leaders provide individuals with a sense of purpose [40], gradually making it the responsibility and obligation of employees to accomplish their goals. According to regulatory focus theory, this change in perception will enhance prevention focus by increasing the excluded employee’s focus on obligation [43]. 

In addition, because of goal congruence, visionary leaders place greater emphasis on the collective role to enhance team performance. Excluded employees will be more concerned about feeling safe in the collective. This is because, in order to accomplish a good vision, the individual must have relaxed thought and a secure environment in the collective in order to influence prevention focus [16]. Rather than perceiving the future as disconnected or distant, visionary leaders help employees recognize the close connection and relevance of their present actions to future aspirations. Expectations and visions for the future are closely linked [16]. By instilling a sense of direction about what the future may bring and what specific future efforts should be made towards, visionary leaders play a controlling role [43]. Therefore, visionary leadership will guide employees in the exclusion of trouble in a timely manner and guide them to reduce resource depletion. This indicates to us that visionary leadership can also increase prevention focus and encourage employees to pay attention to the gap between the current situation and the ideal, motivating them to avoid resource losses [14]. Thus, the following subhypothesis is proposed:

**H3b:**  *Visionary leadership moderates the relationship between workplace ostracism and prevention focus. The higher the degree of visionary leadership, the stronger the positive relationship between workplace ostracism and prevention focus*.

### 2.5. The Moderated Mediation Model

Based on our proposed expectations, we expect that visionary leadership will moderate the mediating role of promotion focus and prevention focus in the relationship between workplace ostracism and proactive behavior, as outlined in the following subhypotheses:

**H4a:**  *Visionary leadership moderates the mediating role of promotion focus in the relationship between workplace ostracism and proactive behavior. When visionary leadership is high, the indirect effect is stronger*.

**H4b:**  *Visionary leadership moderates the mediating role of prevention focus in the relationship between workplace ostracism and proactive behavior. When visionary leadership is high, the indirect effect is stronger*.

**Figure 1:**
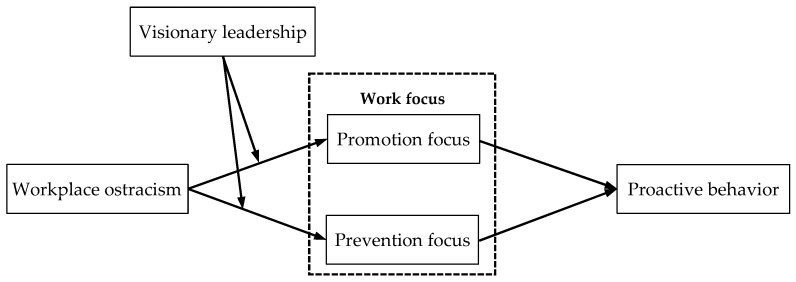
Theoretical model.

Our theoretical model is shown in Figure 1.

## 3. Methods 

### 3.1. Sample and Procedure 

This study employed a multi-time-point questionnaire survey and collected data from employees in various industries in China, specifically in Shandong, Guangdong. These companies are mainly engaged in sales and retail. We first contacted the managers of these companies and explained to them the study we were conducting. After the managers agreed to carry out this study, we carried out the research during tea breaks and regular meetings. At time node T1, a total of 396 questionnaires were distributed to employees, and 357 valid questionnaires were collected, which assessed variables such as demographic information, workplace ostracism, and visionary leadership. After a two-week interval, at time node T2, the same participants were administered another questionnaire, which measured variables including promotion focus, prevention focus, and proactive behavior. A total of 325 valid matched questionnaires were obtained, resulting in an effective recovery rate of approximately 82.0%.

Regarding the sample characteristics, the gender distribution indicated that 49.5% of respondents were male. In terms of age, 54.2% of participants were under the age of 30. In terms of educational attainment, 64.9% of respondents held undergraduate degrees, while 28.6% were graduate students or above. In terms of work experience, 47.7% of participants had worked for less than 1 year, and 48.6% had worked for 2–5 years. We used the structural equation model (SEM) and PROCESS program to analyze the data through MPLUS 8.6 and SPSS 27 to test our hypothesis.

### 3.2. Measures

The scale used in this study is the five-point Likert scale, ranging from “1” to “5”, where 1 means “completely disagree” and 5 means “very agree”. 

*Workplace ostracism*. This variable was measured using a scale developed by Ferris et al., (2008) with a total of 10 statements, such as “In the daily office, others will ignore me” [25]. In this study, the Cronbach’s coefficient was 0.93.

*Work focus*. The measurement of this variable was carried out by adopting the scale developed by Neubert et al., (2008), with a total of 18 items. The first nine items measure prevention focus, and the last nine items measure promotion focus with statements such as “My work focus is affected by the clear picture I desire” and “At work, I am motivated by my hopes and ambitions” [44]. In this study, the Cronbach’s coefficients of prevention focus and promotion focus were 0.77 and 0.78, respectively. 

*Proactive behavior*. The measurement of this variable was carried out by adopting the scale developed by Su and Li (2018), with a total of 9 items, such as “I will take the initiative to complete its own work in a better way” and “I can take the initiative to think about ways to improve its own work” [35]. In this study, the Cronbach’s coefficient was 0.87. 

*Visionary leadership*. The measurement of this variable was carried out by adopting the dimension of vision in the scale developed by Li and Shi (2005), with a total of 6 items, such as “Leaders can point out the goal and direction of struggle for employees” [45]. The Cronbach’s coefficient was 0.84. 

*Control variables*. According to a previous study, it is considered that employee demographic variables may be related to employee proactive behavior [31]. Based on this, this study treats the genders, ages, working times, and education levels of the participants as control variables.

### 3.3. Data Analyses 

Statistical analysis was performed using AMOS 29, SPSS 27, and MPLUS 8.6. First of all, we used AMOS 29 to conduct a confirmatory factor analysis. Then, we adopted SPSS 27 to conduct a descriptive statistical analysis. Further, MPLUS 8.6 was used to verify the mediation path analysis of the data. Finally, we used the PROCESS program in SPSS 27 software to test the moderating effect and moderated mediating effect of the model. 

### 3.4. Confirmatory Factor Analysis

Before testing our hypotheses, we conducted a confirmatory factor analysis (CFA) using AMOS 29 to assess the discriminant validity of our model. Table 1 presents the results of the CFA, which aimed to examine the distinctiveness of the study variables. It can be seen in Table 1 that the five-factor model has the best fitting index (*χ*^2^ = 1352.312, *df* = 815, CFI = 0.914, RMSEA = 0.045, GFI = 0.840, TLI = 0.905).

### 3.5. Common Method Bias

In the research design procedure, in order to reduce the occurrence of common method bias, the questionnaire questions used in this study are unambiguous and easy to understand, and the questionnaires were collected anonymously in multiple locations. In view of the fact that all of the measurement items in this study were filled out by the employees themselves, there is a homologous influence, so the influence of the common method bias was tested using a Harman single-factor test after data collation. All the items of the five variables were merged into one variable, and the factor analysis was performed without rotation. It was found that there was neither a single factor nor a common factor that could explain most of the measurement variance variation (the variance interpretation rate of the first factor was only 15.124%, far lower than 40%); in order to further verify whether there is a common method bias, a two-factor model was established. After adding the common method factor, the fitting indexes of the model did not significantly improve (regarding △CFI = 0.0527 and △TLI = 0.0487, the change did not exceed 0.1; regarding ΔRMSEA = 0.0087, the change did not exceed 0.05) [46]. The above methods indicate that there is no serious common method bias in this study.

### 3.6. Descriptive Statistics and Correlation Coefficient 

The mean, standard deviation, and correlation analysis results of the variables in this study are shown in Table 2. It can be seen in Table 2 that workplace ostracism is significantly positively correlated with prevention focus (r = 0.135, *p* < 0.05) and promotion focus (r = 0.216, *p* < 0.01). There was a significant positive correlation between prevention focus and proactive behavior (r = 0.214, *p* < 0.01) and a significant positive correlation between promotion focus and proactive behavior (r = 0.235, *p* < 0.01). This provides a preliminary basis for this study to further verify the research hypothesis.

### 3.7. Hypothesis Testing 

On the basis of controlling the four demographic factors, namely gender, age, working time, and education level, of the research sample, Mplus 8.6 was used to test the mediating effect of the model. In order to improve the accuracy of the analysis, self-sampling was carried out, and the bootstrap value was set to 2000. The direct effect and mediating effect of the model were tested by a path analysis, and the results are shown in Table 3.

In terms of direct effects, H1a and H1b state that workplace ostracism will have a positive impact on promotion focus and prevention focus. The results show that it has a significant positive effect on promotion focus (β = 0.169, *p* < 0.01) and prevention focus (β = 0.07; *p* < 0.05).

In the mediating effect test, drawing on the results of Wen and Ye (2014) on the mediating effect analysis [47], the bootstrap method was used to directly test the coefficient product to verify the mediating effect. First of all, the indirect effect between the variables of “workplace ostracism → promotion focus → proactive behavior” is significant (β = 0.027; *p* < 0.05), and the 95% confidence interval does not include 0, indicating the mediating role of promotion focus. Similarly, prevention focus also plays a mediating role. The “Workplace ostracism → prevention focus → proactive behavior” path shows a significant positive correlation in the data (β = 0.014, *p* < 0.05), and the 95% confidence interval does not contain 0, so H2a and H2b are verified, which confirms that promotion focus and prevention focus have a mediating effect between workplace ostracism and proactive behavior. 

This study used the PROCESS program compiled by Hayes to verify the moderating role and the moderated mediation effect. The 95% confidence interval was set, and the bootstrap method was used to carry out sampling 5000 times. After controlling for the genders, working times, ages, and education levels of the employees, the moderating effects of the high and low values of the visionary leadership evaluated by the employees were determined and are shown in Table 4. The interaction between workplace ostracism and visionary leadership has a significant positive impact on employees’ promotion focus (β = 0.197; *p* < 0.01). It can be seen in Table 4 that when employees feel a higher level of visionary leadership, promotion focus increases after encountering workplace ostracism (β = 0.257 **; 95% CI = [0.158, 0.357]). When employees feel a lower level of visionary leadership, workplace ostracism has no significant effect on employees’ promotion focus (β = 0.008; 95% CI = [−0.122, 0.138]). Therefore, visionary leadership can effectively moderate the positive relationship between workplace ostracism and employees’ promotion focus. Thus, H3a is supported.

**Figure 2:**
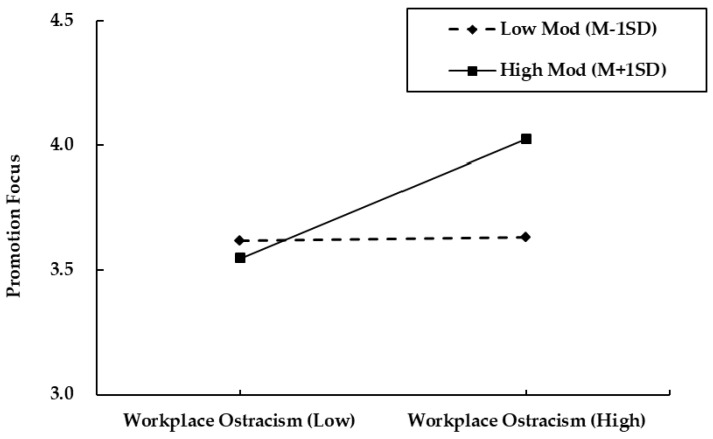
The moderating effect of visionary leadership on the relationship between workplace ostracism and promotion focus.

In order to more clearly show the moderating effect of visionary leadership, this study takes the high standard deviation and low standard deviation of the mean value of the moderating variable, visionary leadership, as the standard of grouping adjustment. We drew an adjustment effect diagram to depict the relationship between promotion focus and workplace ostracism. Specifically, as shown in Figure 2, visionary leadership positively regulates the relationship between workplace ostracism and promotion focus, and employees who perceive high-level visionary leadership are more likely to increase promotion focus after encountering workplace ostracism; for employees who perceive lower levels of visionary leadership, the impact on promotion focus is not obvious after encountering workplace ostracism. H3a is therefore verified.

H3b proposes that visionary leadership moderates the relationship between workplace ostracism and prevention focus. However, through the analysis of the PROCESS program developed by Hayes, the interaction between workplace ostracism and visionary leadership has no significant effect on employees’ prevention focus (β = 0.072; 95% CI = [−0.01, 0.155]), so H3b is not verified.

The results of the moderated mediation effect analysis are shown in Table 5. When the level of visionary leadership is high, the indirect effect of workplace ostracism on employees’ proactive behavior through promotion focus is significant (β = 0.049, SE = 0.015, and 95% CI = [0.022, 0.082]); when the level of visionary leadership is low (one standard deviation below the mean), the indirect effect of workplace ostracism on employees’ proactive behavior through promotion focus is not significant (β = 0.002, SE = 0.011, and 95% CI = [−0.023, 0.023]), and this difference is significant (β = 0.048, SE = 0.017, 95% CI = [0.018, 0.086]). Therefore, H4a is supported. Given that H3b did not receive support, it is evident that H4b is also not supported.

## 4. Discussion 

**Figure 3:**
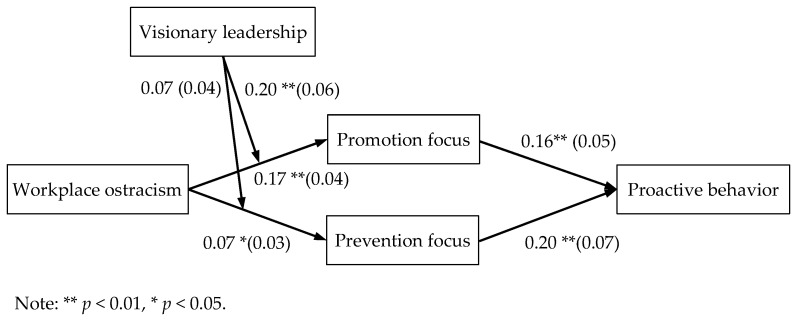
The results of the hypothesis test.

This study concludes that workplace ostracism has a positive impact on employees’ proactive behavior through the mediating role of work focus, specifically promotion focus or prevention focus. However, previous studies generally believe that workplace ostracism has negatively affected employees [1,2,3]. However, these results fail to explain the efforts of some employees to change the status quo after experiencing workplace ostracism. The conclusion of this study is contrary to that of Liu et al., (2015) [5], which is helpful to understand the influence of workplace ostracism on employees’ psychology and behavior in academic and management practices. This study also shows that visionary leadership is a boundary condition influencing employees’ proactive behavior. When employees perceive high-level visionary leadership, they are more likely to increase their promotion focus in response to workplace ostracism, leading to enhanced proactive behavior. However, the moderating role of visionary leadership in the relationship between workplace ostracism and prevention focus is not significant. This unsupported hypothesis shows that visionary leadership can give employees faced with workplace ostracism more power and confidence to exhibit a preference for approach rather than avoidance. Research shows that visionary leadership can improve creativity and team performance [16,17]. The results of this study are in line with the role of visionary leadership in employees and work teams. The results of the hypothesis test are shown in Figure 3.

### 4.1. Theoretical Implications 

First, this study expands on the outcomes of workplace ostracism. We challenge the conventional belief that exclusion only leads to negative outcomes [3,48,49,50]. Although the negative results caused by workplace ostracism are the most common and influential, they are not conducive to understanding the mechanism of workplace ostracism regarding organizations and employees. This controversy is similar to the idea that employees who suffer from workplace gossip may take positive and negative actions to deal with it [51,52]. By demonstrating that employees who experience workplace ostracism exhibit higher levels of proactive behavior, we provide empirical evidence that being excluded from social interactions can motivate individuals to take proactive measures in their work. Based on this, this study provides theoretical and empirical bases for organizations and individuals to deal with workplace ostracism [1], which enriches the research on workplace ostracism.

Second, by showing that workplace ostracism can benefit proactive behavior via work focus, we provide further empirical evidence that promotion focus and prevention focus can exist and increase at the same time [22,23]. Studies have also confirmed that workplace ostracism reduces proactive behavior by reducing organizational self-esteem [5]. This is mainly because the negative experience brought about by workplace ostracism inhibits employees’ organizational self-esteem. Employees are no longer engaged in proactive behavior for the purpose of self-verification. In this study, workplace ostracism is regarded as an available tool. The information it embodies stimulates work focus from two dimensions (the opportunity to show self-worth and the need for security) [21,22], thereby increasing proactive behavior. This also reflects the promotional effect of work focus on employees under stress. Moreover, Higgins (1997) proposed that there are two ways to form work focus: long-term personal traits and situational-induced states [12]. Previous studies have paid more attention to work focus as a stable personal trait that can regulate employees’ behavior while ignoring the path of situational induction. We treat promotion focus and prevention focus as psychological states and show that they can be mechanisms linked workplace ostracism and proactive behavior. Based on this, we provide more theoretical and empirical evidence for the regulatory focus theory.

Finally, we found that visionary leaders have a greater moderating effect on promotion focus than on prevention focus. A good vision can indeed be effectively communicated by a leader to an employee, thus motivating the employee to take positive steps to satisfy the desire for success [16,17,53]. In the face of negative pressures like workplace ostracism, the communication, motivation, planning, and inclusion that visionary leadership encompasses are key factors in helping employees enhance their self-worth [16]. But for employees committed to preventing future negative outcomes, the negative impact of rejection still outweighs the security that comes from leadership. In other words, visionary leadership inspires employees to grow more than it provides a sense of security. The state of promotion focus is more likely to change than that of prevention focus. Based on this, we determined under what circumstances visionary leadership will have an impact on employees.

### 4.2. Practical Implications 

First, organizations should recognize the positive potential of workplace ostracism. While workplace ostracism is often associated with negative outcomes [3,48], this study sheds light on its potential positive impact on employees’ proactive behavior. This is similar to the idea that workplace ostracism will also increase innovation performance [7]. However, it is important to note that this does not imply that workplace ostracism should be endorsed or encouraged. Instead, organizations should take proactive measures, such as increasing regulatory focus, to overcome the negative effects of ostracism and leverage the potential positive outcomes. This study helps managers understand why some employees are not greatly negatively affected by workplace ostracism. Managers can identify which employees who suffer from workplace ostracism should be given priority through the conclusions of this study. 

Second, organizations should prioritize the cultivation of employees’ work focus states to unlock the positive potential inherent in workplace ostracism. Employees with promotion focus pay attention to shaping the perfect self and realizing their purpose; employees with prevention focus pay attention to responsibilities and obligations, what they should accomplish, and what others expect [20]. Our study reveals that the mediating effect of promotion focus is stronger compared to prevention focus. This finding underscores the importance of employees directing their attention towards attaining positive goals rather than adopting a defensive stance. To capitalize on this insight, managers should focus on fostering a work environment that encourages employees to strive for personal and professional growth. This, in turn, can drive proactive behavior and improved performance.

Lastly, organizations should invest in developing and nurturing visionary leadership among managers and supervisors. Visionary leaders have the ability to inspire and motivate employees [41], especially in the face of workplace ostracism. As a visionary leader, M.S. Swaminathan has demonstrated the indispensable role of long-term goals and visions in personal and employee development [53]. Our research indicates that high-level visionary leadership increases employees’ promotion focus in response to workplace ostracism, leading to enhanced proactive behavior. However, visionary leadership’s regulatory role in the relationship between workplace ostracism and prevention focus is not significant. This suggests that visionary leadership primarily benefits employees with promotion focus rather than those with prevention focus.

### 4.3. Limitations and Future Research Directions 

Firstly, existing research shows that workplace ostracism will reduce employees’ proactive behavior through the self-verification process [5]. However, our research challenges this view that workplace ostracism promotes proactive behavior through work focus. But the question we have not answered is as follows: when do employees increase their proactive behavior and when do they decrease their proactive behavior after experiencing workplace ostracism? The coping process of employees after experiencing workplace ostracism shows that there may be a contradictory relationship between workplace ostracism and employees’ proactive behavior [1,5]. Future research can explore whether there is a certain degree of an inverted-U relationship between workplace ostracism and employees’ proactive behavior. A theoretical model can also be designed to explore the double-edged sword effect of workplace ostracism on employee proactive behavior.

Secondly, there may be common method biases in this study. Although we controlled the data acquisition process to avoid the influence of common method biases [54], for example, two time points were used for measurement and an anonymous investigation. However, variables such as workplace ostracism, promotion focus, prevention focus, and proactive behavior are all obtained through employees’ self-reports, so there may still be potential common method biases in this study. Since workplace ostracism is a continuous and changing phenomenon in the workplace [1,25], the experience sampling method can be used to measure the degree of workplace ostracism experienced by employees. Future research can also use a variety of data sources to study the results of workplace ostracism. In addition, this study was carried out in enterprises in China, and the results may be limited. Evidence from enterprises in China may not be fully applicable to other countries. In the future, researchers can explore the differences in the influence of workplace ostracism on employees in the cross-cultural background.

Finally, this study proves the moderating effect of visionary leadership between workplace ostracism and promotion focus, but the moderating effect between workplace ostracism and prevention focus is not significant. This result is inconsistent with previous studies to some extent [15,16]. Although this shows that visionary leaders motivate employees to grow more than provide a sense of security, we only obtained preliminary evidence. In the future, researchers can further study the mechanism of visionary leadership on employees’ psychology and behavior to solve this problem. In the future, researchers can also study the broader boundary conditions between workplace ostracism and work focus.

## 5. Conclusions

This study concludes that workplace ostracism positively impacts employees’ proactive behavior through the mediating role of work focus, specifically promotion focus or prevention focus. Previous studies generally view workplace ostracism as having negative effects on employees, but these results do not account for the efforts of some employees to change the status quo after experiencing ostracism. Differing from Liu et al., (2015) [5], our findings enhance the understanding of workplace ostracism’s influence on employees’ psychology and behavior in academic and management practices. Additionally, this study highlights that visionary leadership serves as a boundary condition influencing employees’ proactive behavior. When employees perceive high levels of visionary leadership, they are more likely to increase their promotion focus in response to workplace ostracism, leading to enhanced proactive behavior. However, visionary leadership does not significantly moderate the relationship between workplace ostracism and prevention focus, suggesting that it empowers employees to prefer approach strategies over avoidance.

## Figures and Tables

**Table 1:** Confirmatory factor analysis results.

Model	Combination	*χ* ^2^	*df*	GFI	TLI	CFI	RMSEA
Five-factor model	WO, Pro, Pre, PB, VL	1352.312	815	0.840	0.905	0.914	0.045
Four-factor model	WO, VL, Pro+Pre, PB	2108.120	854	0.745	0.788	0.799	0.067
Three-factor model	WO+VL, Pro+Pre, PB	2868.270	857	0.648	0.660	0.678	0.085
Two-factor model	WO+VL, WF+PB	3104.639	859	0.630	0.622	0.640	0.090
One-factor model	WO+VL+WF+PB	4741.053	860	0.452	0.347	0.378	0.118

Note: N = 325; WO = workplace ostracism; Pro = promotion focus; Pre = prevention focus; PB = proactive behavior; VL = visionary leadership; WF = work focus.

**Table 2:** Descriptive statistics and correlations.

Variable	*Mean*	*SD*	1	2	3	4	5	6	7	8	9
1. Gender	1.500	0.501	1								
2. Age	1.490	0.570	−0.203 **	1							
3. Education level	3.200	0.614	0.052	−0.177 **	1						
4. Work time	1.840	0.919	−0.133 *	0.575 **	−0.124 *	1					
5. Workplace ostracism	3.039	0.928	−0.068	0.057	−0.119 *	0.011	1				
6. Prevention focus	4.092	0.482	0.037	−0.099	−0.016	−0.043	0.135 *	1			
7. Promotion focus	3.708	0.726	−0.047	−0.039	0.028	−0.052	0.216 **	0.269 **	1		
8. Proactive behavior	4.040	0.571	−0.022	−0.065	0.008	−0.073	−0.007	0.214 **	0.235 **	1	
9. Visionary leadership	3.989	0.632	−0.036	−0.054	0.053	−0.008	0.031	0.216 **	0.150 **	0.511 **	1

Note: N = 325; ** *p* < 0.01 and * *p* < 0.05.

**Table 3:** Path analysis: direct and mediating effects.

Effect	Path	β	SE	95% CI
Direct effect	Workplace ostracism → Prevention focus	0.070	0.030	[0.012, 0.127]
	Workplace ostracism → Promotion focus	0.169	0.043	[0.088, 0.251]
	Prevention focus → Proactive behavior	0.200	0.067	[0.066, 0.329]
	Promotion focus → Proactive behavior	0.162	0.050	[0.068, 0.267]
	Workplace ostracism → Proactive behavior	−0.046	0.038	[−0.123, 0.030]
Mediating effect	Workplace ostracism → Prevention focus → Proactive behavior	0.014	0.008	[0.002, 0.014]
	Workplace ostracism → Promotion focus → Proactive behavior	0.027	0.011	[0.010, 0.027]

**Table 4:** Analysis results of moderating effect (promotion focus).

Effect Relationship	Workplace Ostracism × Visionary Leadership → Promotion Focus	
	Moderating Effect	95% CI
High level of visionary leadership	0.257 **	[0.158, 0.357]
Low level of visionary leadership	0.008	[−0.122, 0.138]

Note: N = 325; ** *p* < 0.01.

**Table 5:** Conditional indirect effect of workplace ostracism on proactive behavior.

Effect Type		β	SE	95% CI
Workplace ostracism × Visionary leadership → Promotion focus → Proactive behavior	Visionary leadership (+1SD)	0.049	0.015	[0.022, 0.082]
	Visionary leadership (mean)	0.025	0.010	[0.007, 0.048]
	Visionary leadership (−1SD)	0.002	0.011	[−0.023, 0.023]
	High–low (difference)	0.048	0.017	[0.018, 0.086]

## Data Availability

Data are contained within the article.
